# Factors affecting hand cosmesis and the aesthetic impact of surgery on congenital hand differences in Finland

**DOI:** 10.1177/17531934221139698

**Published:** 2022-11-30

**Authors:** Noora N. Nietosvaara, Antti J. Sommarhem, Antti Stenroos, Aarno Y. Nietosvaara, Petra Grahn

**Affiliations:** 1 Department of Pediatric Orthopedics and Traumatology, University of Helsinki and Helsinki University Hospital, Finland; 2 Department of Surgery, Central Hospital of South Karelia, Lappeenranta, Finland; 3 Department of Orthopedics and Traumatology, University of Helsinki and Helsinki University Hospital, Finland; 4 Department of Pediatric Surgery, Kuopio University Hospital, University of Eastern Finland, Finland

**Keywords:** Upper extremity deformities, aesthetics, hand, child, disability evaluation

## Abstract

We assessed the appearance and cosmetic impact of surgery in congenitally different hands in Finland. A questionnaire was sent to 1165 respondents (786 female) with a mean age of 33 years (range 3–84). Participants were shown nine image pairs and seven pairs of pre- and postoperative images twice in a random order and asked to choose the more cosmetically pleasing one. We found that the appearance and number of fingers had an important aesthetic role, with higher number and more normal appearing digits consistently scoring higher than its counterpart (range 59–99%). Postoperative appearances were perceived as better than preoperative ones in syndactyly (98%), thumb duplication (92%), cleft hand (93%) and radial dysplasia (99%). Toe transfer and pollicization had little impact on cosmesis. This study demonstrated that surgery could improve cosmesis in congenitally different hands and overall, most respondents prefer an appearance that is as close as possible to normality. **Level of evidence:** IV

## Introduction

The reported incidence of upper limb deficiencies is 5–20 per 10,000 births (Ekblom et al., 2010; Giele et al., 2001; Koskimies et al., 2011). In Scandinavia 30–50% of children born with an upper limb difference were treated surgically (Koskimies-Virta et al., 2020; Ekblom et al., 2010), with the most common surgical procedures being removal of supernumerary digits and syndactyly separation (Koskimies-Virta et al., 2019). Surgery is ideally performed during the first years of life but almost always before school age (Koskimies-Virta et al., 2019). Although the main goal of surgery is traditionally to prioritize function over appearance, cultural- and surgeon-related differences exist with regards to these priorities (Little and Cornwall, 2016;  Singer et al., 2014; Yen et al., 2006). Additionally, surgery that improves function may positively impact appearance (Kozin, 2012, Libberecht et al., 2011).

No widely accepted means of quantifying the appearance of the congenitally malformed hand exist. Aesthetic outcome after surgery has been evaluated in a few studies (Goldfarb et al., 2007; Libberecht et al., 2011; Staines et al., 2005; Yen et al., 2006), but the measurement of these were usually fairly subjective and reported, as ‘improved, good, or satisfying’ according to surgeons, parents or both. Malformations can cause functional, cosmetic and psychological disability, which may partly be related to stress from peers (Andersson et al., 2011; Bickham et al., 2017; McDougall et al., 2021). For this reason, improving the hand’s appearance has been reported as more important than functional improvement for children and their parents’ following surgery by some authors (Libberecht et al., 2011). A previous study on the general perception of congenitally malformed hands found that appearance is perceived poorly in hands with three or fewer fingers, several abnormally developed parts, cleft hand or any other clearly visible deformity (Nietosvaara et al., 2021). Surgery may improve the appearance of congenitally different hands and bring them closer to normal, but it also creates visible scars that may impact appearance negatively.

This study aims to further analyse factors affecting the general perception of the appearance of hand differences and to get an understanding of which aspects of a deformed hand are perceived as positive or negative in Finland. The second aim is to analyse the overall aesthetic impact of congenital hand surgery.

## Methods

### Data collection

A digital questionnaire was developed to study the general perception of different congenital hand differences. Photographs of children’s hands were selected from the picture library of Helsinki Children’s Hospital, a tertiary paediatric hand unit, to obtain as broad a spectrum of paediatric hand differences as possible (Table 1). The study was divided into two parts; the first aimed to analyse what specific aspects of the malformed hand were considered to have a greater impact on the aesthetic outcome (deformity level, number, shape, finger length and presence/absence of nails). In order to study this, the conditions of transverse longitudinal deficiency and different configurations of symbrachydactyly, ulna dysplasia and amniotic band syndrome were chosen. The second part of this study was to analyse whether surgery improved the aesthetic appearance of hands in general. Pre- and postoperative photographs of different conditions were included to assess the effect of surgery including thumb duplication, syndactyly (simple complete), thumb hypoplasia, cleft hand, radial longitudinal deficiency and symbrachydactyly (Blauth, 1967; Manske et al., 2017; Oberg et al., 2010; Woodside and Light, 2016). For radial longitudinal deficiency, postoperative photographs were included to show the results of two different surgical procedures (radialization and second toe metatarsophalangeal joint transfer) (Buck-Gramcko, 1985; Vilkki, 1998) (Table 1). The background of all photographs was standardized with the hand size and skin tone digitally edited to be uniform, and the hand positioned similarly (dorsum of hand upwards, wrist in neutral position, fingers extended).

**Table 1.** Description of the conditions and types of surgery for comparison.

Pair numbers	Conditions for comparison
Appearance of conditions	
Pair 1	Transverse longitudinal deficiency at wrist level versus symbrachydactyly with nubbins
Pair 2	Transverse longitudinal deficiency at wrist level versus monodactylous symbrachydactyly
Pair 3	Monodactylous versus bidactylous symbrachydactyly
Pair 4	Bidactylous symbrachydactyly versus two-finger ulnar longitudinal deficiency
Pair 5	Two- versus three-finger ulnar longitudinal deficiency
Pair 6	Three- versus four-finger ulnar longitudinal deficiency
Pair 7	Four-finger ulnar longitudinal deficiency versus normal hand
Pair 8	Amniotic band syndrome A versus B
Pair 9	Amniotic band syndrome B versus C
Impact of surgery on appearance	
Pair 10	Correction of syndactyly (simple complete)
Pair 11	Correction of thumb duplication (Wassel II)
Pair 12	Closure of cleft hand
Pair 13	Pollicization for thumb hypoplasia (Blauth V)
Pair 14	Second toe transfer for symbrachydactyly
Pair 15	Distraction and radialization for radial longitudinal deficiency (Type IV)
Pair 16	Distraction and radialization versus second metatarsophalangeal joint transfer for radial longitudinal deficiency (Type IV)

The selected photographs of the hands for the first part were divided into nine pairs, and the pre- and postoperative images forming another seven for the second part. These 16 pairs of images (Table 1) were shown to the participants twice in random order via a computer tablet at the time of recruitment, or through an email link. Participants were asked to choose the more aesthetically pleasing image in each set of pairs by pressing a ‘thumbs up’ icon under each photograph. If the participants gave a different answer the second time the image was shown, their answers were regarded as inconsistent.

### Recruitment of participants

Participants were recruited at two randomly chosen public schools (one primary and one high school), public areas in two Finnish cities (Helsinki and Kuopio), and from Helsinki and Kuopio University hospitals. Age, gender and profession (adults) or level of education (age under 18) were registered. The reported professions included medical doctors (*n* = 447), university students (*n* = 226), nurses (*n* = 79) and participants with miscellaneous professions (*n* = 214). Of the participants under 18 years of age, 178 were school children and 199 high school students. Participants were asked whether they, their children or someone they knew had a limb difference.

### Statistical analysis

A reliability analysis was made using intraclass correlation coefficients based on a two-way mixed-effects model, single rating and absolute agreement to evaluate intra-rater reliability. The internal consistency of inter-rater reliability was estimated using Cronbach’s alpha coefficient. The frequency distribution of the categorical variables was compared between the groups with the chi-squared test and Mann–Whitney *U*-test for continuous variables. We acquired descriptive statistics using cross-tabulations and nominal variables were presented in counts and percentages. In the case of missing values, the percentages were compared against the valid cases. A multivariate risk factor analysis for choosing the right pair was done using binary logistic regression. Odds ratios with 95% confidence intervals (CI) were calculated for each possible risk factor. A pairwise deletion was conducted for the analyses in case of missing values. *P*-values less than 0.05 were considered to be statistically significant.

## Results

**Figure 1.**
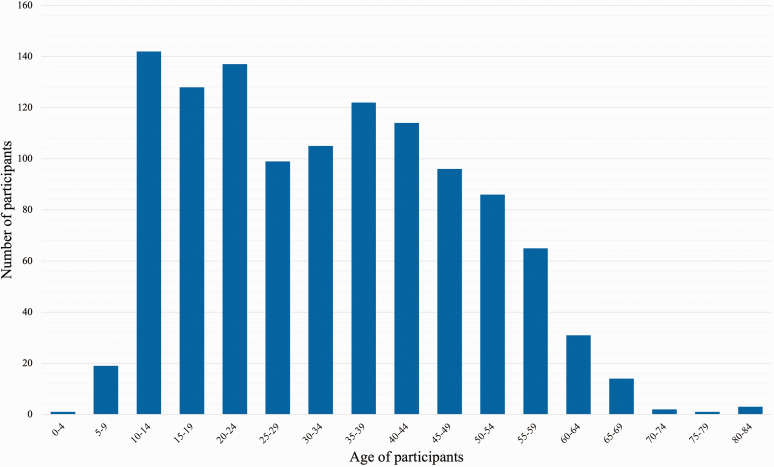
Age distribution of participants with a mean age of 33.

A total of 1165 (786 female) persons were recruited. The mean age of the participants was 33 (range 3–84, SD 15.5) (Figure 1). Of all respondents, 14 had a congenital upper limb malformation, 20 had children with one and 185 knew someone with one.

**Figure 2.**
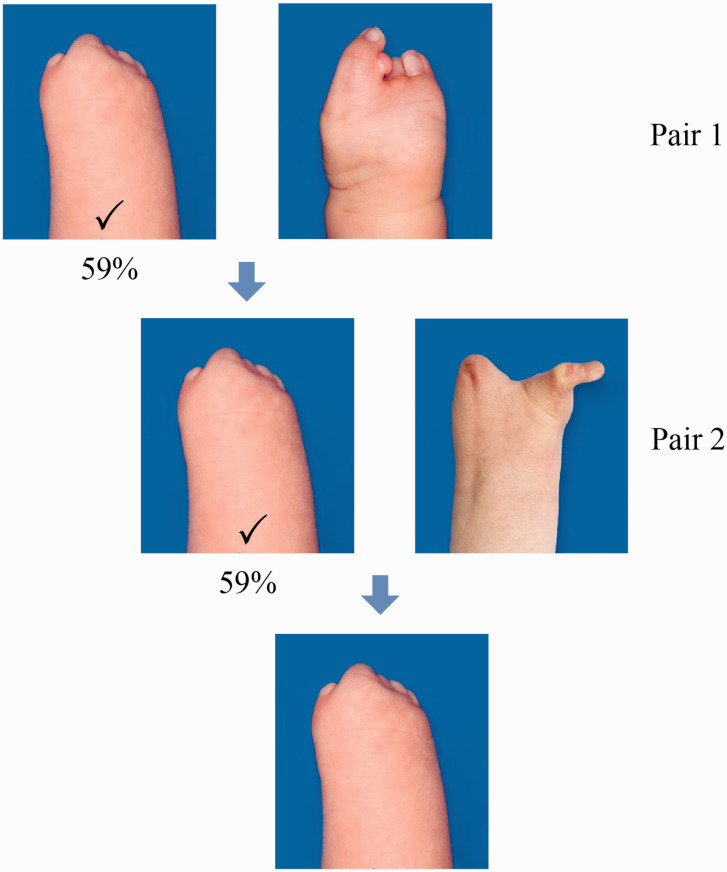
Results of comparison of pairs 1 and 2 with most (59%) preferring a transverse longitudinal deficiency over symbrachydactyly with nubbins (32%, Pair 1) and transverse longitudinal deficiency (59%) over monodactylous symbrachydactyly (32%, Pair 2). For Pair 1, 9% of participants gave an inconsistent answer, and for Pair 2, 9%.

**Figure 3.**
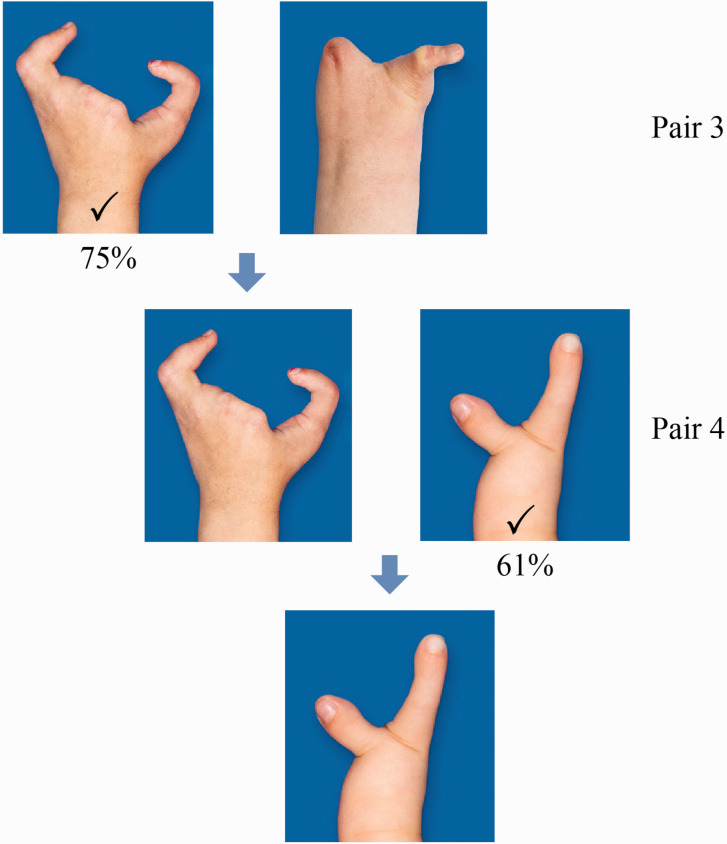
Results of comparison of Pairs 3 and 4 with most (75%) preferring a bidactylous symbrachydactyly over monodactylous (15%, Pair 3) and a two-finger ulnar longitudinal deficiency (61%) over bidactylous symbrachydactyly (24%, Pair 4). Ten per cent of participants gave an inconsistent answer for Pair 3, and 15% for Pair 4.

**Figure 4.**
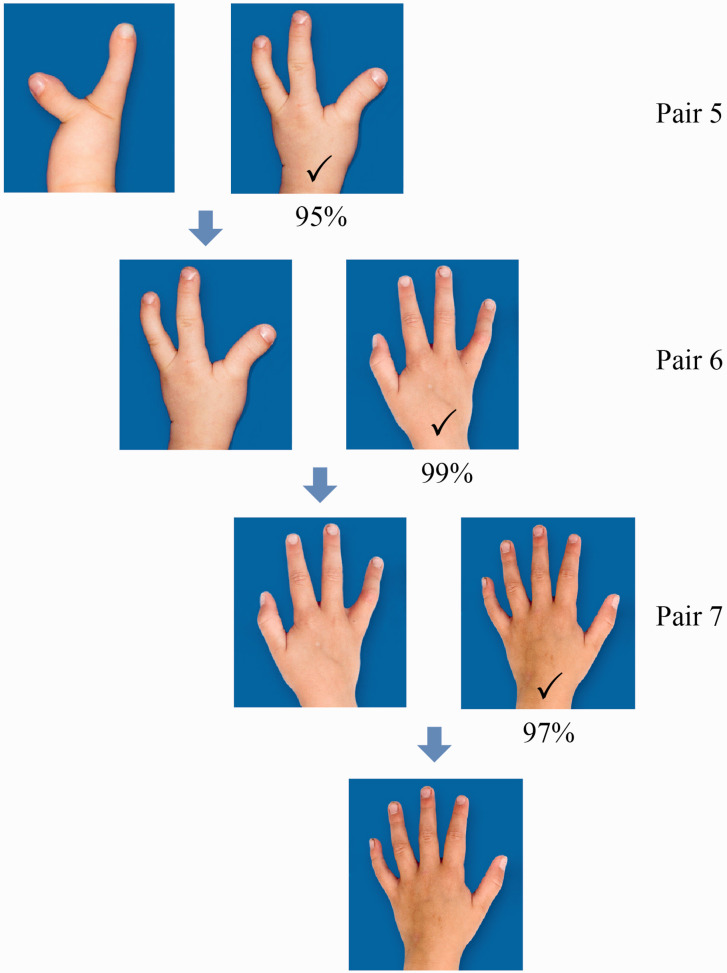
Results of comparison of hands with different degrees of ulnar longitudinal deficiency (Pairs 5, 6 and 7). Most preferred a tree-finger (95%) over a two-finger hand (1%, Pair 5), a four-finger (99%) over a three-finger hand (0%, Pair 6) and a normal (97%) over a four-finger hand (1%, Pair 7). The percentage of participants who gave inconsistent answers was 4% for Pair 5, 1% for Pair 6 and 2% for Pair 7.

**Figure 5.**
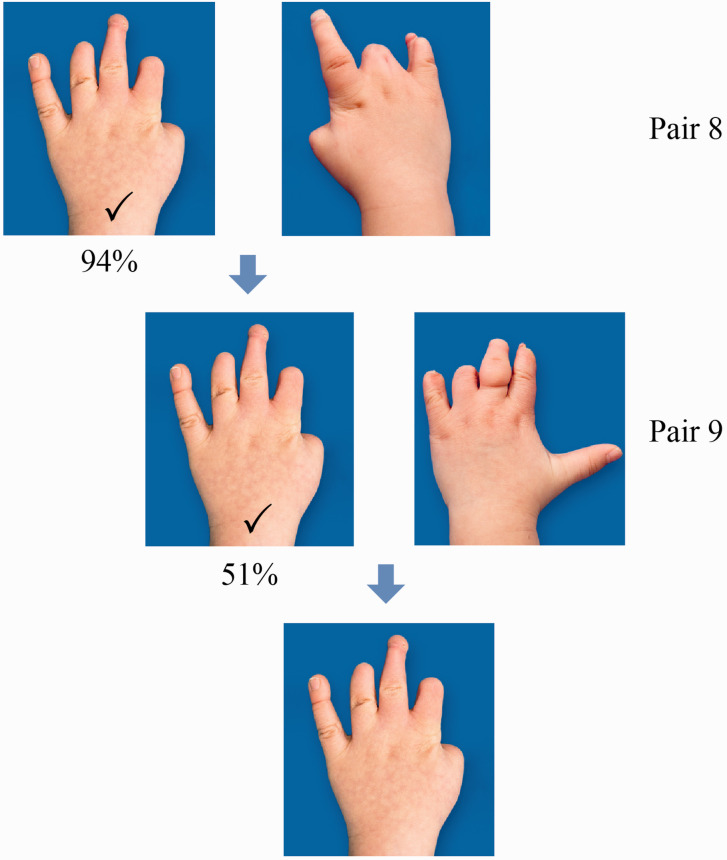
Results of comparison of different amniotic band syndrome (ABS) hands (Pairs 8 and 9). Most preferred the ABS hand with four digits (94%) instead of one with two (2%, Pair 8). Fifty-one per cent of participants chose the ABS hand with 4 ‘more normal’ appearing digits without a thumb to the hand with four more affected digits and a thumb (33%, Pair 9). The percentage of participants who gave inconsistent answers was 4% for Pair 8 and 17% for Pair 9.

Most respondents found transverse longitudinal deficiency at wrist level more aesthetically pleasing than symbrachydactyly with nubbins or monodactylic symbrachydactyly (Figure 2). A two-finger ulnar longitudinal deficiency hand was perceived as more attractive than its bidactylous symbrachydactylous counterpart, which again was considered significantly more appealing than monodactylous symbrachydactyly (Figure 3). The closer the resemblance in appearance to a normal looking hand (length and number of digits) the more unanimous was the score of the respondents, with a normal hand and a four-finger ulnar longitudinal deficiency hand scoring the highest (Figure 4). Also, there were more who found the amniotic syndrome hand with a normal little finger more aesthetically pleasing than the hand with a thumb but abnormal little finger with 17% giving inconsistent answers (Figure 5).

**Figure 6.**
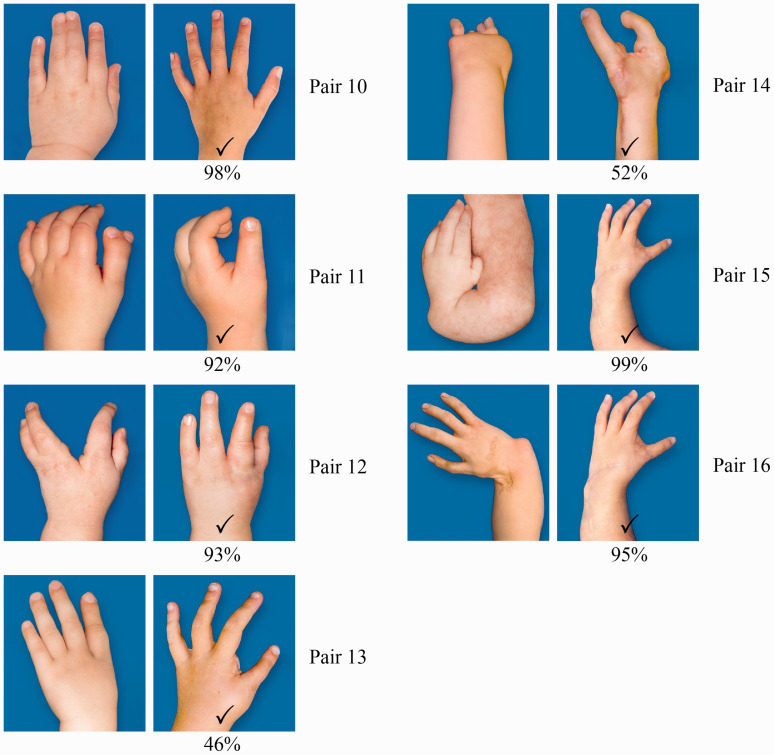
Results of the pre- and postoperative hands (Pairs 10–16). The percentage of participants who changed their answer was 1% in Pair 10, 6% in Pair 11, 3% in Pair 12, 15% in Pair 13, 12% in Pair 14, 1% in Pair 15 and 3% in Pair 16.

The postoperative image of syndactyly, thumb duplication, cleft hand and radial longitudinal deficiency was perceived as better than the preoperative image with a high inter-rater agreement (Figure 6). The image of a symbrachydactylous hand after toe-to-hand transfers was slightly better, than its preoperative image, with 12% giving an inconsistent answer. A hand with thumb hypoplasia (Blauth V) was found almost equal in appearance before and after pollicization, with the highest number of inconsistent answers (15%). In radial dysplasia hand the postoperative image of radialization was superior to the metatarsophalangeal joint transfer, with only 3% inconsistent answers. The percentage of inconsistent answers varied between 1–17%.

In the multivariate analysis of the different subgroups (age, sex, profession, relation or patient with congenital hand anomaly), there was no statistically significant difference regarding answer distribution for Pairs 1, 2, 3, 7, 9, 10, 12, 15 or 16 (Table 1). There was a higher inter-rater agreement in the support for the winning image within the subgroup of medical doctors with regards to two-finger ulnar longitudinal deficiency (Pair 4, OR 1.15, CI 0.7–1.7, *p* = 0.001) and amniotic band syndrome (Pair 8, OR 1.9, CI 0.5–6.5, *p* = 0.03). The same was found in children 10 to 14 years of age for three-finger ulnar longitudinal deficiency (Pair 5, OR 23.3, CI 1.8–48.6, *p* = 0.05), and participants 20 to 89 years of age for four-finger ulnar longitudinal deficiency (Pair 6, OR 5.2, CI 1.6–21.6, *p* = 0.026) as well as postoperative cleft hand (Pair 12, OR 0.3, CI 0.1–1.8, *p* = 0.02). When looking at group inter-rater, children under the age of 10 and females had a slightly higher agreement for the preoperative image of the thumb duplication (Pair 11) in comparison to the other groups (OR 0.3, CI 0.19–0.9, *p* = 0.03 and OR 0.48, CI 0.2–1.1, *p* = 0.03). The same was found in children 10–14 years of age for the preoperative radial longitudinal deficiency (Pair 15, OR 0.8, CI 2.7–3.6, *p* = 0.01), and in children under 10 years of age with regards to the toe-to-hand transfer (Pair 16, OR 8.2, CI 0.9–29.4, *p* = 0.03).

Disagreement between winning image per group was found regarding the four-finger hand before and after pollicization (Pair 13), where medical doctors and participants aged 20–89 predominantly chose the postoperative image (OR 1.8, CI 1.2–2.8, *p* < 0.001 and OR 0.5 CI 0.2–0.8, *p* = 0.01). Similar disagreement was found for the pre- and postoperative images of the toe-to-hand transfer (Pair 14), where medical doctors (OR 2.3, CI 1.5–3.4, *p* < 0.001) and male participants (OR 1.2, CI 0.8–1.7, *p* = 0.04) preferred the postoperative image. Other respondents predominantly chose the preoperative image. Responses by participants who had or whose child had an upper limb difference or who knew someone with an upper limb difference did not differ from the rest of the respondents.

## Discussion

In this study, we aimed to understand how congenitally different hands are perceived and what impact surgery has on the appearance of these hands. The first part of the study analysed different factors affecting the appearance of congenital hand differences. The deformity level (wrist, metacarpal, finger) was not considered as important for appearance as abnormally developed parts was. Transverse longitudinal deficiency was considered more aesthetically pleasing than a monodactylous hand with a malformed thumb, and two-finger ulnar longitudinal deficiency was better perceived than a bidactylous symbrachydactyly hand, possibly suggesting that normal-looking fingers with nails are more important than the deformity level. Our pairwise comparison further confirmed the importance of normal-shaped fingers; nubbins, underdeveloped or misshaped fingers were regarded inferior to hands with an abnormal number but more normal-looking digits. Nails are perceived as essential for the normal appearance of fingers, while nails in the wrong places seem to negatively impact appearance, which could be why a hand with nubbins and nails was perceived as cosmetically less pleasing than a nearly identical hand without nails. The results of this study further strengthen earlier findings that the thumb does not affect appearance more than any other finger, and that the total number of normal-appearing digits matters (Nietosvaara et al., 2021).

Surgery can improve the cosmetic appearance of most common congenital hand differences, as shown in the second part of this study shows. The most probable reason is the restoration of hand symmetry, which can be observed after the removal of supernumerary digits, separation of syndactyly and closure of cleft. However, in our study neither toe-to-hand transfer nor pollicization clearly improved the hand’s appearance. Bellew et al. (2011) have reported contradictory results, with most patients (20/22) and parents (17/22) being more satisfied with the hand’s appearance postoperatively after undergoing toe-to-hand transfer. In the same study, most parents (19/21) and patients (18/19) thought surgery had improved their overall hand function. Despite the potential functional improvement gained from toe-to-hand transfers, the outcome can be limited by the postoperative aesthetic appearance; toes will always look like toes, especially in a still image, which could explain why only a minority preferred the postoperative appearance. As with toes for digits, pollicized digits for thumbs are longer and narrower than a normal thumb (Goldfarb et al., 2007), which might explain why many of our survey’s participants did not find pollicization to result in a better-looking hand. The results of Bellew et al. (2011) were based on parent and patient reports, which do not necessarily reflect the general population’s viewpoints. Other studies have reported differences in results reported by caregivers compared with laypeople and care providers, with the former often giving better scores (Gkantidis et al., 2013; Goldfarb et al., 2007; Nietosvaara et al., 2021). In this study, we found the answers of caregivers and patients consistent with those of the other participants. Another important consideration is the concept of ‘dynamic cosmesis’ (Bellew et al., 2011), based on the premise that a functional hand attracts less attention. Almost half of the patients in their study reported teasing related to the affected hand, with 60% hiding their hand/s around new people, but psychosocial well-being was reported to be the same or somewhat improved after surgery (Bellew et al., 2011). Reconstructive surgery could, in these cases, be essential for the psychosocial well-being of children with upper limb differences, as suggested by Libberecht et al. (2011).

Responses from medical professionals in our study may be influenced by their belief in what is considered functionally critical for the hand, which could explain why medical doctors chose the thumb over the little finger, preferring the pollicized thumb over the four-digit hand and the double toe-transfer in a symbrachydactylous hand with nubbins. However, even if our risk factor analysis showed a tendency towards some skewness in the answers regarding the participant’s profession, age and gender, the overall results regarding the preferred image only changed with the double toe-to-hand transfer. If medical doctors were excluded, the preferred image was the preoperative symbrachydactylous hand with nubbins. As with all congenital malformations, cosmesis for the patient, parent and bystander is most likely a combination of functional and actual aesthetics, which is why a more reliable result could be achieved using videos rather than still images, as suggested by Kozin (2012) and Bellew and Kay (1999). Although the primary goal of surgery for congenital hand differences aims to improve function, we believe these findings will be helpful from the hand surgeon’s perspective in preoperative planning and counselling for patients and their families. The findings may also justify surgery for minor cosmetic disturbances, even if the function will be unimproved. When comparing Pair 16 to assess the impact of radialization versus the use of the second metatarsophalangeal joint transfer for radial longitudinal deficiency, the majority chose the former. This may be influenced by the residual deviation of the wrist. In our experience, the wrist often ends up in a more deviated position after metatarsophalangeal joint transfer as compared with radialization, even though the amount of wrist motion may be better. In the future, it would be reasonable to conduct a study focusing on both function and appearance as well as possible donor site morbidity.

This study has several limitations. We used sample photographs to represent the conditions, and we are aware the results cannot be applied to all patients with the same condition since every patient’s hand is different. Also, all postoperative images do not necessarily look the same. Moreover, the geographic distribution is limited to the country of Finland, where the religious or cultural backgrounds of participants were not gathered. In some Asian countries, significant effort is put into reconstructing Blauth 3B or 4 thumbs, as having five instead of four digits is culturally more accepted. In Nordic countries, functional improvement is considered the main aim of surgical intervention, whereas surgery based on cultural aesthetics alone is rare. Future studies can include a worldwide study with the cooperation of paediatric hand surgeons from Europe, Africa, Asia-Pacific, North and South America, which may yield interesting results reflecting the different cultural and social considerations.
